# Case Report: Successful treatment of SAPHO syndrome refractory to adalimumab with upadacitinib

**DOI:** 10.3389/fimmu.2026.1818236

**Published:** 2026-04-17

**Authors:** Jinwan Du, Yeshan Li, Shaohui Geng, Chen Li, Shike Shang

**Affiliations:** 1 Department of Rheumatology and Immunology, Liangjiang Hospital of Chongqing Medical University, People’s Hospital of Chongqing Liangjiang New Area, Chongqing, China; 2 School of Chinese Pharmacy, Beijing University of Chinese Medicine, Beijing, China; 3 School of Life Science, Beijing University of Chinese Medicine, Beijing, China; 4 Shanghai Guanghua Hospital of Integrated Traditional Chinese and Western Medicine, Shanghai, China; 5 Department of Traditional Chinese Medicine Rehabilitation, Liangjiang Hospital of Chongqing Medical University, People’s Hospital of Chongqing Liangjiang New Area, Chongqing, China

**Keywords:** adalimumab, case report, JAK inhibitor, SAPHO syndrome, upadacitinib

## Abstract

**Background:**

Synovitis, acne, pustulosis, hyperostosis, and osteitis (SAPHO) syndrome is a rare autoinflammatory disorder characterized by osteoarticular and cutaneous manifestations. While tumor necrosis factor-alpha (TNF-α) inhibitors such as adalimumab are increasingly used for refractory cases, some patients exhibit inadequate response. Janus kinase (JAK) inhibitors have emerged as a potential alternative, but data on the selective JAK1 inhibitor upadacitinib in SAPHO syndrome are lacking.

**Case summary:**

An 18-year-old male presented with a 7-year history of recurrent facial acne since 2016. Whole-body bone scintigraphy revealed focally increased radiotracer uptake in the right clavicle, consistent with SAPHO syndrome. Initial treatment of acne with adapalene gel combined with oral minocycline failed to improve skin symptoms, leading to the decision to ultimately pursue biologic therapy. After four months of adalimumab therapy, the patient initially showed improvement in chest pain and dermatitis but subsequently experienced paradoxical worsening of facial acne accompanied by erythema and pruritus. No improvement was observed after an additional four weeks of continued adalimumab treatment. Following a transition to upadacitinib, facial lesions improved within 4 weeks and achieved marked resolution by 8 weeks.

**Conclusion:**

This report of upadacitinib in SAPHO syndrome demonstrates its rapid and substantial efficacy in a patient refractory to adalimumab. Upadacitinib may represent a promising treatment option for difficult-to-treat SAPHO syndrome, particularly in cases with inadequate response to TNF-α inhibition.

## Introduction

Synovitis, acne, pustulosis, hyperostosis, and osteitis (SAPHO) syndrome is a rare autoinflammatory disorder characterized by a diverse spectrum of dermatological and osteoarticular manifestations (1, 2). The pathogenesis of SAPHO syndrome remains incompletely understood, but various pro-inflammatory cytokines, including tumor necrosis factor-alpha (TNF-α) and interleukin-1 beta (IL-1β), have been implicated in its pathophysiology (1, 2). Treatment options include non-steroidal anti-inflammatory drugs (NSAIDs), analgesics, disease-modifying antirheumatic drugs (DMARDs), biologics, and antibiotics (3, 4).

In recent years, TNF-α inhibitors such as adalimumab have been increasingly used for SAPHO cases refractory to conventional therapies (5–7). However, a subset of patients exhibits primary or secondary failure to TNF-α inhibition, creating an unmet therapeutic need. JAK inhibitors have been applied in SAPHO patients with resistance or intolerance to conventional drugs and biologics (8). The JAK-signal transducer and activator of transcription (STAT) pathway, a ubiquitous intracellular signaling network, mediates the effects of multiple cytokines involved in SAPHO pathogenesis (9, 10). Blocking this pathway has emerged as a potential therapeutic target.

Upadacitinib is an oral JAK inhibitor that selectively inhibits JAK1. While first-generation JAK inhibitors (tofacitinib and baricitinib) have shown efficacy in SAPHO syndrome (11–13), data on selective JAK1 inhibition in this condition are lacking. Herein, we report a case of SAPHO syndrome with inadequate response to adalimumab that achieved remarkable clinical improvement following treatment with upadacitinib.

## Case description

**Figure 1:**
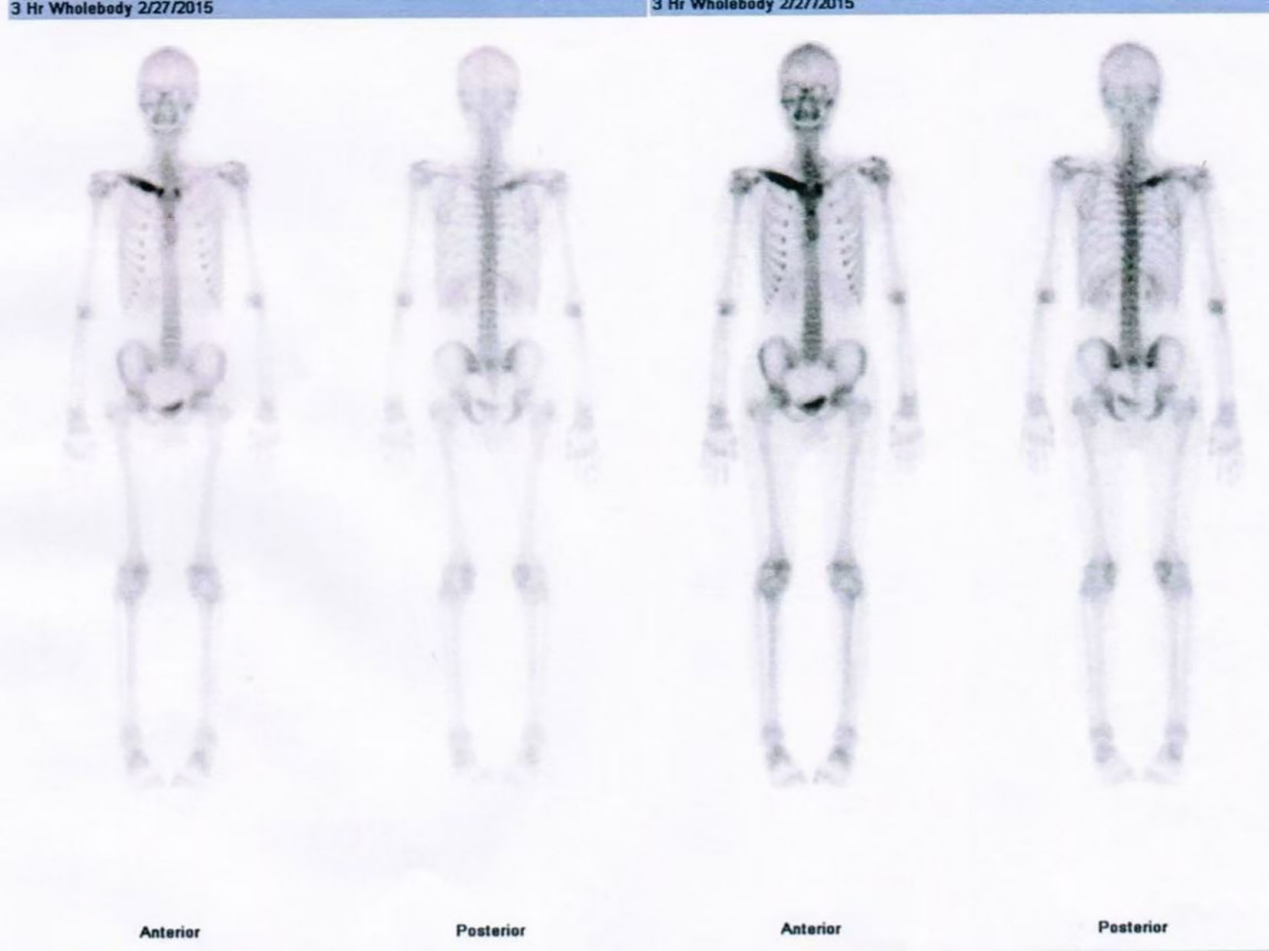
Diagnostic bone scintigraphy in SAPHO syndrome. Anterior and posterior views showing focally increased radiotracer uptake in the right clavicle, consistent with SAPHO syndrome.

**Figure 2:**
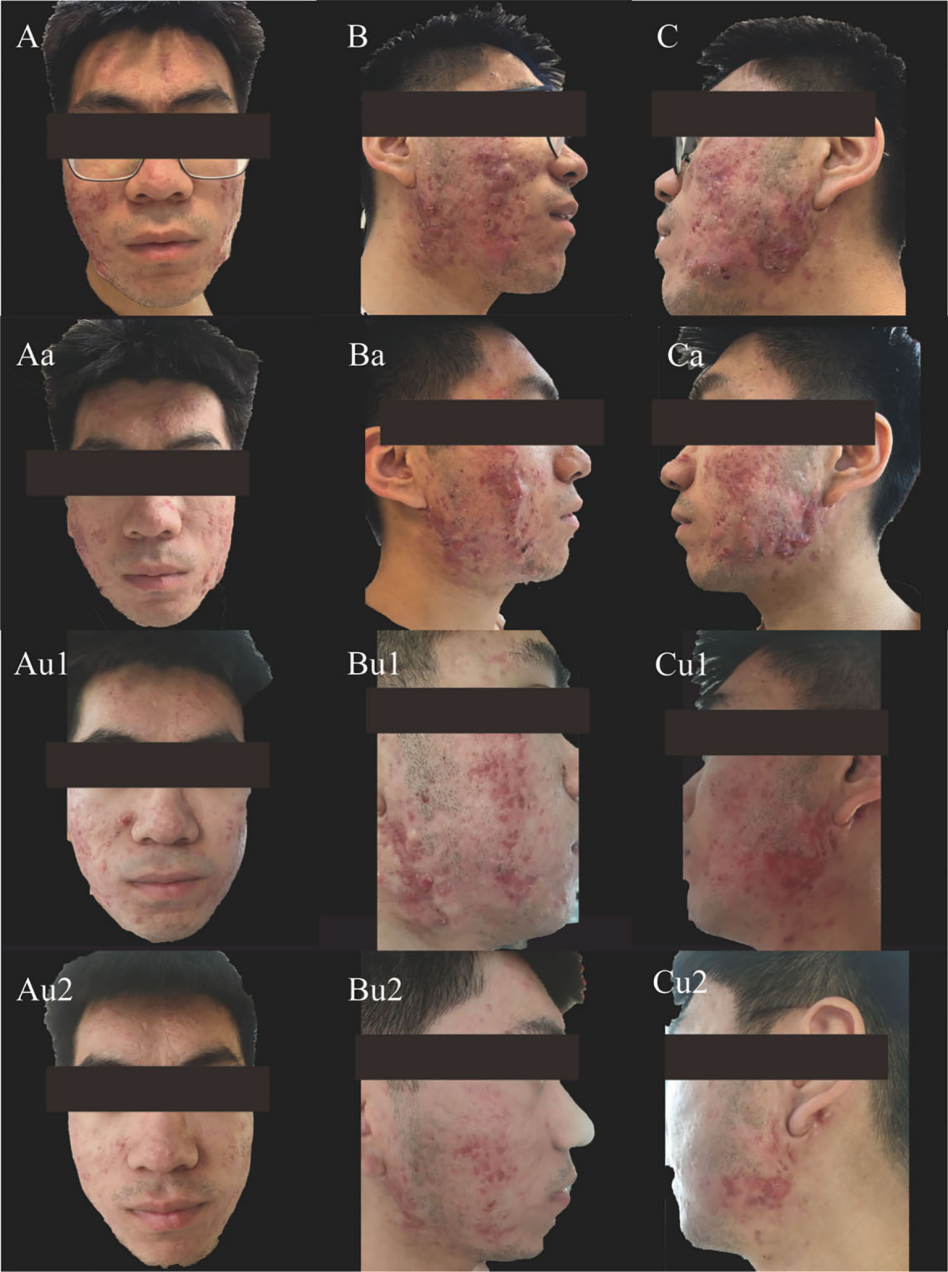
Clinical evolution of facial lesions after switching from adalimumab to upadacitinib. **(A–C)** Facial acne approximately six months after discontinuing adalimumab and prior to treatment with upanotinib. **(Aa–Ca)** No improvement after 4 additional weeks of adalimumab therapy. **(Au1–Cu1)** Marked improvement after 4 weeks of upadacitinib treatment. **(Au2–Cu2)** Near-complete resolution after 8 weeks of upadacitinib treatment.

An 18-year-old male presented with recurrent facial acne that began in 2016 and had recurred repeatedly between 2016 and 2023. The acne was treated with adapalene gel (once daily for three months) in combination with oral minocycline (50 mg once nightly), which provided insufficient control. There was no significant medical, family, or psycho-social history. Laboratory investigations and imaging were conducted. Whole-body bone scintigraphy revealed focally increased radiotracer uptake in the right clavicle (**Figure 1**), consistent with SAPHO (synovitis, acne, pustulosis, hyperostosis, osteitis) syndrome. The acne involved the face, chest, and back, with nodular and cystic lesions on the cheeks, consistent with severe acne (**Figures 2A–C**). The diagnosis was established according to the Kahn diagnostic criteria (2003) (14), based on the characteristic bone scintigraphy finding (“bull’s head” sign) and the presence of severe acne.

Following diagnosis, treatment with subcutaneous adalimumab (80 mg every two weeks) was initiated. After four months, the patient’s chest pain and dermatitis showed significant improvement. Subsequently, however, the facial acne worsened, accompanied by facial redness and itching. No improvement was observed after continuing the same treatment for an additional four weeks (**Figure 2**, Aa-Ca). No new medications were introduced during this period, suggesting that the worsening of facial acne was a paradoxical reaction to adalimumab.

**Figure 3:**
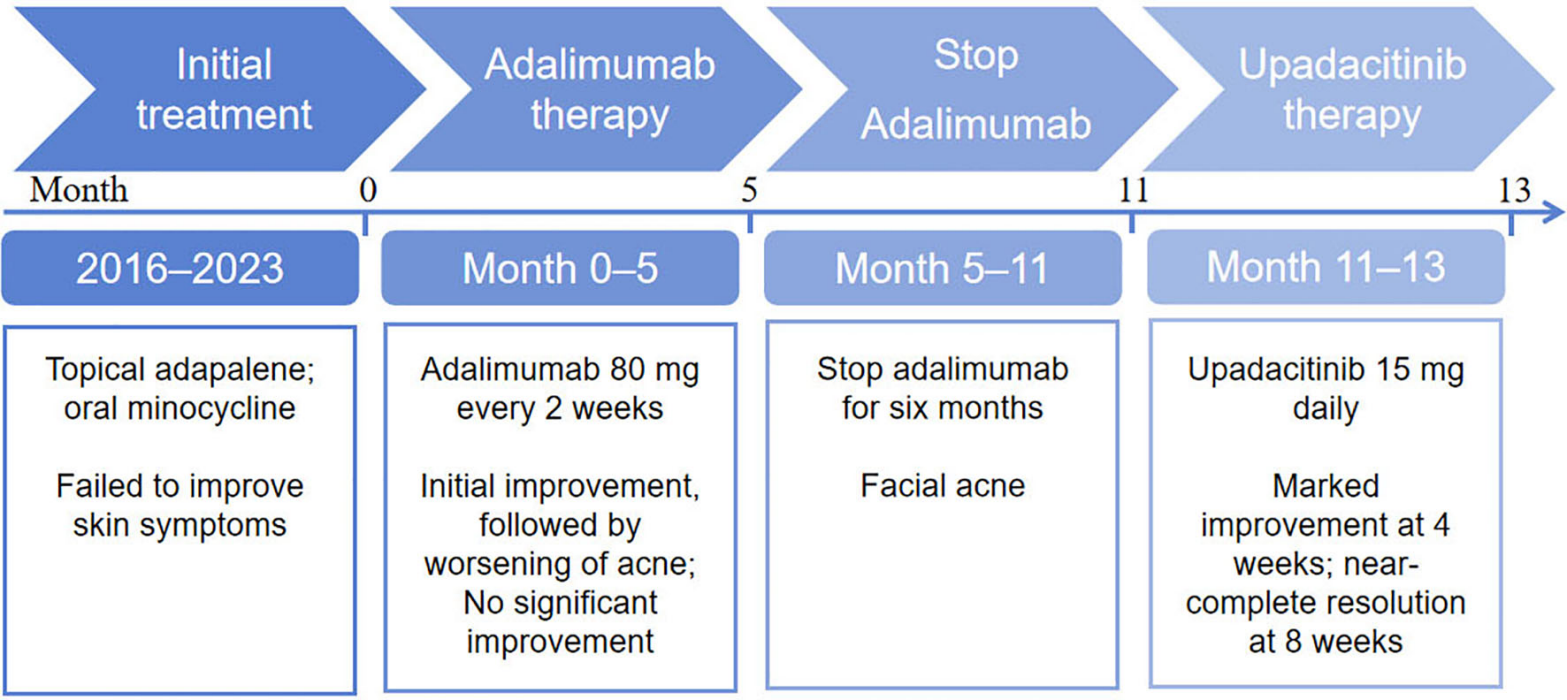
Clinical timeline of the patient. The timeline illustrates the disease course from symptom onset in 2016 to follow-up after upadacitinib treatment.0 indicates the start of treatment with adalimumab; 11 indicates the start of treatment with upanotinib.

Given the inadequate response to adalimumab, therapy was transitioned to oral upadacitinib (15 mg once daily). Four weeks after the transition, facial lesions improved (**Figure 2**, Au1-Cu1). After eight weeks, the facial lesions had resolved markedly (**Figure 2**, Au2-Cu2). Subjectively, the patient reported a significant improvement in his quality of life and expressed high satisfaction with the treatment outcome, highlighting the positive psychological impact of the treatment. The clinical timeline is summarized in **Figure 3**.

## Discussion

Multiple cytokines, including TNF-α, interleukin-1 (IL-1) andinterleukin-17 (IL-17), have been found to mediate the inflammatory response in SAPHO syndrome (8, 9). The JAK-STAT pathway is a ubiquitous intracellular signaling network, and the anti-inflammatory effects of JAK inhibitors are achieved through blockade of multiple cytokines signaling pathways (10). Blocking the JAK-STAT pathway has emerged as a promising therapeutic target for SAPHO syndrome. Several studies have reported that JAK inhibitors are effective in SAPHO syndrome, including refractory cases unresponsive to biologics (11–13). For instance, a case report by Yang Y et al. demonstrated the efficacy of upadacitinib in a patient refractory to secukinumab (an IL-17A inhibitor), improving both joint pain and palmoplantar pustulosis (15). Tofacitinib has also shown effectiveness in difficult-to-treat cases (16). Another report by Jin D et al. described improvement in a SAPHO patient with poor response to conventional drugs and biologics, including TNF-α and IL-17 inhibitors, after combined treatment with guselkumab (an IL-23 inhibitor) and upadacitinib (17). These findings suggest that the efficacy of JAK inhibitors may be individualized, and clinical application should be tailored to the specific patient context. The JAK family consists of four members: JAK1, JAK2, JAK3, and TYK2 (18). JAK inhibitors are a family of small molecules that can block one or more of these four members. Two first-generation JAK inhibitors have been approved for the treatment of inflammatory arthritis: tofacitinib (a JAK1/JAK3 inhibitor) and baricitinib (a JAK1/JAK2 inhibitor) (19, 20). It has been increasingly recognized that certain adverse effects of first-generation JAK inhibitors, such as an increased risk of infections, gastrointestinal inflammation, hematologic abnormalities, and malignancies (21). This has prompted the development of next-generation highly selective inhibitors. Selective JAK inhibitors affect the phosphorylation and activation of different JAKs, thereby blocking the cascade of inflammatory cytokines. To date, most research on chronic inflammatory diseases has focused on preferential inhibition of JAK1 and JAK3, as these JAKs are primarily involved in mediating signals downstream of pathogenic cytokines, while avoiding the potentially harmful effects of JAK2 inhibition (22). Second-generation JAK inhibitors are designed to have selective affinity for one JAK enzyme, minimizing effects on other cytokines with the goal of reducing adverse reactions. Selective inhibitors may provide better control of inflammation while reducing the risk of harm. Upadacitinib is an oral JAK inhibitor that selectively inhibits JAK1. In this report, we treated SAPHO syndrome with upadacitinib in a patient with inadequate response to adalimumab and observed significant improvement in facial lesions. Upadacitinib may represent a therapeutic approach for refractory SAPHO syndrome. However, studies have reported that patients with atopic dermatitis may develop upadacitinib-related acne (23). Therefore, further research is needed to elucidate the mechanisms underlying the improvement or worsening of acne in patients treated with upadacitinib.

Several limitations of this report should be acknowledged. First, as a single case report, the findings cannot be generalized to all SAPHO patients. Second, the follow-up period of eight weeks is relatively short; long-term efficacy and safety of upadacitinib in SAPHO remain to be established. Third, we did not perform mechanistic studies, such as cytokine profiling before and after treatment, which could have provided insights into the immunological changes underlying clinical improvement. Fourth, the off-label use of upadacitinib in SAPHO requires confirmation in larger studies.

## Conclusion

This case provides the evidence that upadacitinib may be a highly effective treatment option for SAPHO syndrome refractory to TNF-α inhibitors. It reinforces the pathogenic role of JAK-STAT signaling in SAPHO and introduces a targeted therapeutic strategy for this challenging disease. Further studies are warranted to confirm these findings and identify optimal patient selection criteria.

## Data Availability

The original contributions presented in the study are included in the article/supplementary material. Further inquiries can be directed to the corresponding authors.
